# Hypoxic preconditioning of human urine-derived stem cell-laden small intestinal submucosa enhances wound healing potential

**DOI:** 10.1186/s13287-020-01662-2

**Published:** 2020-04-06

**Authors:** Xiu-Ru Zhang, Yi-Zhou Huang, Hong-Wei Gao, Yan-Lin Jiang, Jun-Gen Hu, Jin-Kui Pi, An-Jing Chen, Yi Zhang, Li Zhou, Hui-Qi Xie

**Affiliations:** 1 Laboratory of Stem Cell and Tissue Engineering, Orthopaedic Research Institute, State Key Laboratory of Biotherapy and Cancer Center, West China Hospital, Sichuan University and Collaborative Innovation Center of Biotherapy, No.1 Ke-Yuan-Si-Lu, Gao-Peng-Da-Dao, Chengdu, 610041 Sichuan China; 2 Department of Orthopaedics, West China Hospital, Sichuan University, Chengdu, 610041 China; 3 Surgery of Spine and Spinal Cord, Henan Provincial People’s Hospital, Zhengzhou, 450000 China

**Keywords:** Hypoxic preconditioning, Small intestinal submucosa, Urine-derived stem cells, Wound healing

## Abstract

**Background:**

Urine-derived stem cells (USCs) are a valuable stem cell source for tissue engineering because they can be harvested non-invasively. Small intestine submucosa (SIS) has been used as scaffolds for soft tissue repair in the clinic. However, the feasibility and efficacy of a combination of USCs and SIS for skin wound healing has not been reported. In this study, we created a tissue-engineered skin graft, termed the SIS+USC composite, and hypothesized that hypoxic preconditioning would improve its wound healing potential.

**Methods:**

USCs were seeded on SIS membranes to fabricate the SIS+USC composites, which were then cultured in normoxia (21% O_2_) or preconditioned in hypoxia (1% O_2_) for 24 h, respectively. The viability and morphology of USCs, the expression of genes related to wound angiogenesis and reepithelialization, and the secretion of growth factors were determined in vitro. The wound healing ability of the SIS+USC composites was evaluated in a mouse full-thickness skin wound model.

**Results:**

USCs showed good cell viability and morphology in both normoxia and hypoxic preconditioning groups. In vitro, hypoxic preconditioning enhanced not only the expression of genes related to wound angiogenesis (*VEGF* and *Ang*-*2*) and reepithelialization (*bFGF* and *EGF*) but also the secretion of growth factors (VEGF, EGF, and bFGF). In vivo, hypoxic preconditioning significantly improved the wound healing potential of the SIS+USC composites. It enhanced wound angiogenesis at the early stage of wound healing, promoted reepithelialization, and improved the deposition and remodeling of collagen fibers at the late stage of wound healing.

**Conclusions:**

Taken together, this study shows that hypoxic preconditioning provides an easy and efficient strategy to enhance the wound healing potential of the SIS+USC composite.

## Background

Large skin wounds caused by trauma, burns, or chronic diseases remain a significant clinical challenge worldwide [1]. Autologous skin grafts have been frequently used in the clinic, but have some disadvantages, such as limited availability. Therefore, tissue-engineered skin grafts have been developed to repair skin defects [2]. These cell-based therapies have been regarded as promising treatments, since they have improved wound healing in many preclinical studies [3]. However, the search for an ideal cell source is still of great interest because most cell sources, including bone marrow and adipose-derived mesenchymal stem cells (MSCs), require invasive sampling and are impeded by limited supply.

As a relatively new type of adult stem cell, urine-derived stem cells (USCs) were first identified by Zhang et al. in 2008, showing robust proliferation and multilineage differentiation potential [4]. USCs have many advantages for tissue engineering applications: they share biological characteristics with MSCs [5]; can be isolated from autologous urine via a simple, noninvasive, and low-cost approach [6]; and can be obtained from donors regardless of gender, age, or health condition (except for urinary tract infection or anuria) [7]. These features make USCs particularly attractive for creating personalized grafts for tissue regeneration, and as such, a few studies have reported the use of USCs for wound healing [8, 9].

As the main component of the extracellular matrix of human tissues, collagen is broadly used for cutaneous wound healing since it can create a biomimetic microenvironment for tissue regeneration [10]. Small intestinal submucosa (SIS) is a decellularized collagen-based membrane that also contains many other components of an extracellular matrix, especially a variety of growth factors. It has been regarded as a good scaffold for soft tissue regeneration because of good biodegradability, biocompatibility, and the ability to enhance cell adhesion, proliferation, and migration [11, 12].

After injury, the microenvironment of skin wounds is hypoxic due to the disruption of blood vessels and the high consumption of oxygen by the cells around the wounds [13, 14]. The hostile microenvironment of wounds can lead to poor healing outcomes [15–17], such as abnormal structure, disability, and scar formation [18, 19]. Furthermore, it hinders the function of seed cells in tissue-engineered skin grafts, impairing their wound healing ability [20, 21]. Thus, it is necessary to develop easy and applicable methods to improve the repair function of seed cells.

Hypoxic preconditioning has been considered as a simple and effective approach to promote the repair potential of stem cells [22]. It has been shown that short-term exposure of MSCs to hypoxia enhanced cell survival, inhibited cell apoptosis [23], and promoted the expression of growth factors [24]. Furthermore, hypoxia preconditioning has been shown to enhance the therapeutic effects of MSCs in several diseases, such as cerebral ischemia [25] and femoral head osteonecrosis [26]. However, it is still unknown whether hypoxic preconditioning improves the skin wound healing potential of USCs. Herein, we used USCs as seed cells and SIS as a scaffold to create a tissue-engineered skin graft, namely the SIS+USC composite, and determined the effect of hypoxic preconditioning on its wound healing potential in a mouse full-thickness skin wound model.

## Material and methods

### Isolation and culture of human USCs

USCs were obtained from five healthy male adult donors (20–35 years old) using methods described previously [4, 5]. Two hundred milliliters of fresh urine was collected from each donor. The samples were treated with 1% penicillin and streptomycin (Gibco, USA) and centrifuged for 10 min at 1500 rpm. The supernatant was discarded and the sediment was washed twice in phosphate-buffered saline (PBS, ZSGB-BIO, China). The cells from the pellet were seeded in 6-well plates and cultured in cell culture medium comprised of 50% Keratinocyte Serum-Free Medium (KSFM, Gibco, USA), 33.75% Dulbecco’s modified Eagle medium (DMEM, Gibco, USA), 11.25% Ham’s F-12 Nutrient Mixture (Gibco, USA), and 5% fetal bovine serum (FBS, Gibco, USA) supplemented by 5 ng/mL epidermal growth factor (EGF, Gibco, USA), 50 ng/mL bovine pituitary extract (Scienceu, USA), 0.4 μg/mL hydrocortisone (Sigma, USA), 5 μg/mL transferrin (Sigma, USA), 5 ng/mL bovine insulin (Sigma, USA), 0.18 mM adenine (Sigma, USA), 2 nM 3,3,5-triiodo-l-thyromine (Sigma, USA), 100 units/mL penicillin (Gibco, USA), and 100 μg/mL streptomycin (Gibco, USA). The medium was first changed 7 days after cell seeding and the non-adherent cells were removed. Culture medium was refreshed twice per week. When the cells reached 80% confluence, they were passaged at a ratio of 1:3. Cells at passage 3 were used in the following experiments.

### Characterization of human USCs

#### Osteogenic differentiation

The cells were grown to 80% confluence in a 6-well plate and incubated with osteogenic induction medium (Gibco, USA), which was refreshed every 3 days. After 28 days of induction, the cells were stained with Alizarin Red S (Sigma, USA) to detect calcified extracellular matrix.

#### Adipogenic differentiation

The cells were grown to 80% confluence in a 6-well plate and incubated with adipogenic medium (Gibco, USA) that was changed every 3 days. After 21 days of induction, the cells were stained with Oil Red O (Sigma, USA) to visualize lipid vacuoles.

#### Flow cytometry analysis

The cells were incubated with 3% bovine serum albumin (Sigma, USA) for 30 min to block nonspecific antigens. Then, they were incubated with the following monoclonal antibodies (BD, USA) for 30 min: CD29-APC, CD34-APC, CD44-FITC, CD45-PE, CD73-PE, CD90-FITC, and HLA-DR-FITC. After incubation, the cells were washed with PBS and analyzed in a flow cytometry analyzer (Beckman Cytomics FC500, Beckman Coulter, USA).

### Preparation of the SIS+USC composites

The SIS membranes were prepared according to the method described in our previous studies [27, 28]. Briefly, fresh porcine jejunums were harvested from market pigs (around 100 Kg at 6 months) within 3 h of sacrifice, cut into pieces of approximately 10 cm in length, and washed thoroughly with a saline solution. The serosa, tunica muscularis, and tunica mucosa were mechanically removed, and then the submucous membranes were degreased by immersing the samples in an organic solution containing methanol and chloroform (1:1, V/V) for 12 h. After degreasing, the membranes were decellularized by sequential incubation in 0.05% trypsin for 12 h and 0.5% sodium dodecyl sulphate for 4 h. Then, the samples were disinfected by soaking in 0.1% peracetic acid, rinsed with a saline solution, and freeze-dried at − 70 °C for 48 h by using a lyophilizer (CHRIST, GAMMA 2–16 LSC, Germany). Finally, the obtained SIS membranes were cut into circular membranes with a diameter of 10 mm, sealed into hermetic packages, and sterilized by using ethylene oxide gas.

Before cell seeding, the SIS samples were washed twice with cell culture medium. Then, 1 × 10^6^ USCs were seeded on the surface of each SIS membrane, and the SIS+USC composites were divided into the following groups: (1) the SIS+USC (N) group, in which the composites were cultured in a normoxia incubator (21% O_2_, 5% CO_2_) (Thermo, USA) for 24 h and (2) the SIS+USC (H) group, in which the composites were cultured in a hypoxia incubator (1% O_2_, 5% CO_2_) (Thermo, USA) for 24 h.

### Characterization of the SIS+USC composites

#### Live/dead staining

The SIS+USC composites were incubated with 1 μM calcein AM (Sigma, USA) and 1 μM propidium iodide (Roche, USA) for 30 min in the dark, washed 3 times with PBS, and then visualized in a confocal microscope (A1RMP+, Nikon, Japan).

#### Hematoxylin and eosin (H&E) staining

The SIS+USC composites were fixed in 4% paraformaldehyde (PFA, Beyotime, China) for 24 h. All samples were washed 3 times with PBS, dehydrated using graded ethanol, and embedded in paraffin. Sections (4 μm thick) were mounted on slides for H&E staining, and the staining results were observed with a fluorescence microscope (Zeiss, Germany).

#### Scanning electron microscope (SEM) observation

The specimens were fixed in 2.5% glutaraldehyde (Sigma, USA) for 24 h at 4 °C, dehydrated with graded ethanol, lyophilized at − 80 °C for 24 h, and vacuum-dried overnight. Then, they were sputtered with gold and observed with a scanning electron microscope (JEOL, Japan).

#### Real-time polymerase chain reaction (RT-PCR)

RNA samples from each group (*n* = 4) were extracted using TRIZOL reagent (Life Technologies, USA), and cDNA was synthesized using an RNA PCR kit (Life Technologies, USA). The primers for RT-PCR are listed in Additional file 1: Table S1. The reaction conditions were as follows: 2 min at 95 °C, followed by 50 cycles of 95 °C for 15 s and 60 °C for 30 s. Gene expression was calculated by comparative ΔΔCq method. Briefly, mean Cq values were normalized to the internal GAPDH and the difference was defined as ΔCq. The comparative gene expression level was expressed as 2^−ΔΔCq^.

#### Enzyme-linked immunosorbent assay (ELISA)

The cell culture supernatants of each group (*n* = 4) were separately aspirated for the measurement of EGF, vascular endothelial growth factor (VEGF), and basic fibroblast growth factor (bFGF) using the ELISA kits according to the manufacturer’s instructions (Ray biotech, USA).

### Wound healing potential of the SIS+USC composites in vivo

#### CM-Dil labeling

To track the fate of USCs in vivo, the cells were labeled with CM-Dil (C7000, Invitrogen, USA), which can be retained in the labeled cells throughout the fixation of tissue samples, permeabilization, and paraffin embedding [29, 30]. After labeling, the cells were used to prepare the SIS+USC composites, as described in the above section. Then, the SIS+USC composites were used for the following animal studies.

#### Animal experiments

Thirty-two male BALB/c nude mice weighted at 18–24 g were anesthetized with pentobarbital sodium at a dose of 75 mg/kg. Two full-thickness excisional skin wounds with an 8-mm diameter were created on the back of each mouse. The wounds were divided into one of the following groups: (1) the control group, in which the wounds were treated with saline; (2) the SIS group, where the wounds were covered with SIS membrane alone; (3) the SIS+USC (N) group, in which the wounds were treated with the SIS+USC (N) composites; and (4) the SIS+USC (H) group, in which the wounds were covered with the SIS+USC (H) composites (*n* = 16 wounds per group). In groups 3 and 4, the SIS+USC composites were applied on the wounds directly, and the cell sheets formed on the surface of composites were in direct contact with the wound beds. A silicon splint ring (inner diameter, 10 mm; outer diameter, 16 mm) was placed on the skin around the wound to prevent skin contraction [31].

On days 0, 4, 7, 14, and 21 post-treatment, the wound area was calculated by tracing the wound margins of each mouse. The wound healing rate was calculated with the following formula:

$$ \mathrm{Wound}\ \mathrm{healing}\ \mathrm{rate}=\left(\mathrm{initial}\ \mathrm{wound}\ \mathrm{area}-\mathrm{residual}\ \mathrm{wound}\ \mathrm{area}\right)/\mathrm{initial}\ \mathrm{wound}\ \mathrm{area}\times 100\%. $$

#### Histological analysis

At each time point, eight mice were euthanized by over-dose injection of 8% (w/v) chloral hydrate on days 4, 7, 14, and 21 after surgery. Skin samples were harvested for HE and Sirius red staining.

Briefly, the samples were fixed in 4% PFA for 24 h before being embedded in paraffin. Sections (4 μm thick) were mounted on slides for histological staining. To visualize the formation of new tissue, H&E staining was performed, and the staining results were captured using a fluorescence microscope (Zeiss, Germany).

Sirius red staining was performed to observe the deposition of collagen fibers in the wound beds. The results were observed in a polarized microscope (Leica, Germany), followed by quantitative evaluation with a digital image analysis software (Image-Pro plus software 6.0, Bethesda, USA). The randomly selected images (3 images per specimen) with a general amplification of × 400 were digitalized in 3264 × 2448 pixels and a resolution of 300 dpi. The areas occupied by types I and III collagen were measured, and their ratios to the total area of the image were analyzed.

#### Immunofluorescence staining

Paraffin-embedded samples were dewaxed with xylene, endogenous peroxidase was blocked with 3% hydrogen peroxide, and sections were rinsed with PBS and blocked with 10% goat serum (Beyotime, China) for 20 min. Subsequently, primary antibodies of rabbit monoclonal anti-cytokeratin 14 antibody (ab181595, 1:1000, Abcam) or goat monoclonal anti-CD31 antibody (AF3628, 1:100, R&D) were incubated with the samples at 4 °C overnight. The slides were then washed with PBS and incubated with goat anti-rabbit secondary antibody (1:250, Jackson ImmunoResearch) or rabbit anti-goat secondary antibody (1:250, Jackson ImmunoResearch) for 1 h at 37 °C. Staining results were observed with a fluorescence microscope (Zeiss, Germany), and the images were quantitatively evaluated by a digital analysis software (Image-Pro plus 6.0, Bethesda, USA). For each specimen, 3 randomly selected images were used to analyze the vascularization and reepithelialization of wounds.

### Statistical analysis

Statistical analyses were conducted using SPSS 20.0 software (Chicago, IL, USA). Data was presented as the mean ± standard deviation. Normal distribution of all variables was tested, and if all variables met normal distribution, the statistical trends were analyzed by one-way ANOVA with a post hoc Tukey’s test; otherwise, the Kruskal-Wallis test was used. Statistical significance was set at *p* < 0.05 versus the indicated group.

## Results

### Characterization of human USCs

**Fig. 1:**
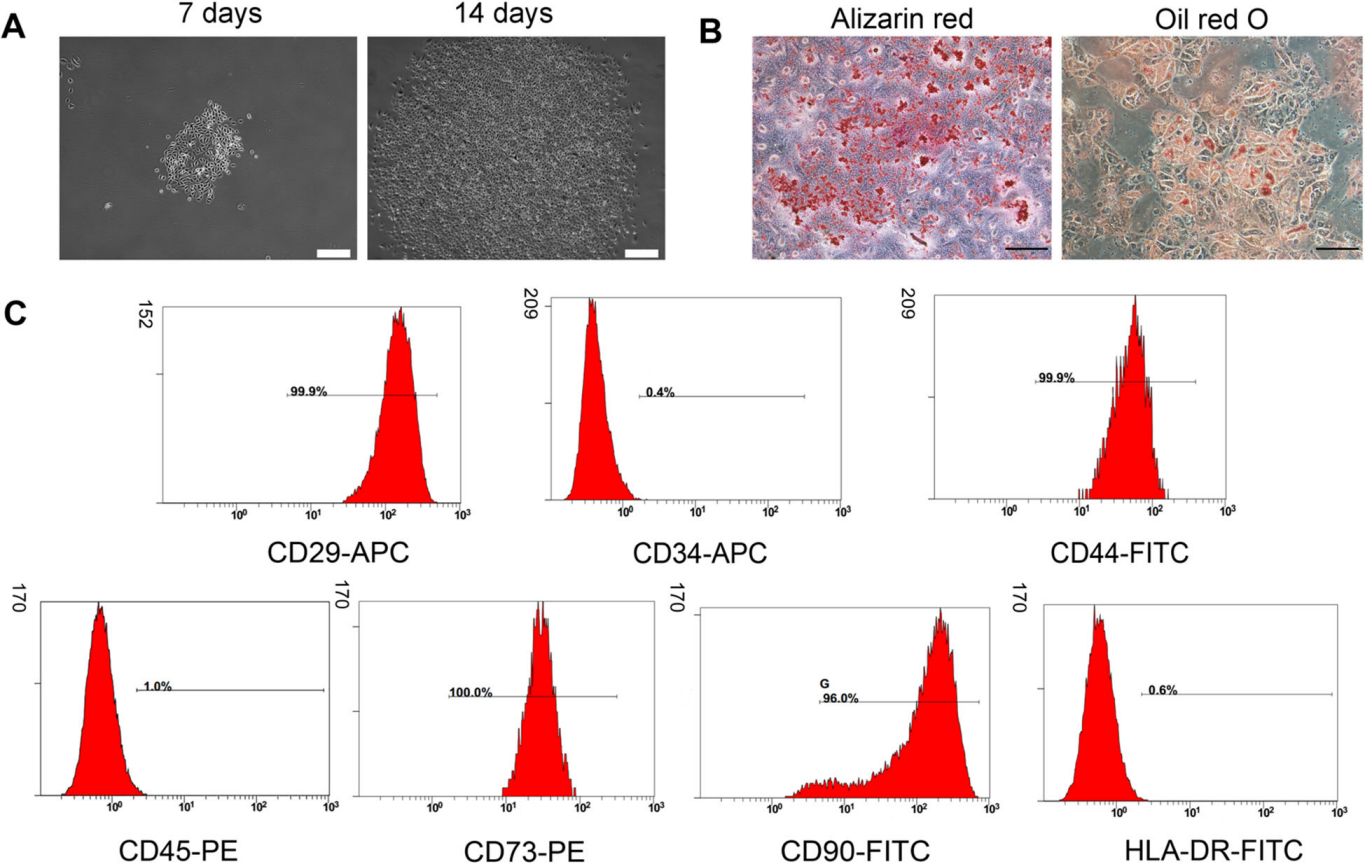
Characterization of human USCs. **a** The morphology and proliferation of USCs. Scale bar, 200 μm. **b** The osteogenic and adipogenic differentiation of USCs. Scale bar, 100 μm. **c** The cell surface marker expression of USCs

As shown in Fig. 1a, USCs had a “rice grain” appearance and robust proliferation capability. Similar to our previous study [5], they can undergo osteogenic and adipogenic differentiation after induction in vitro, as indicated by positive staining for Alizarin Red and Oil Red O, respectively (Fig. 1b). Furthermore, the phenotype of USCs was similar to that of MSCs [5]: positive for CD29, CD44, CD73, and CD90 but negative for CD34, CD45, and HLA-DR (Fig. 1c).

### Characterization of the SIS+USC composites

**Fig. 2:**
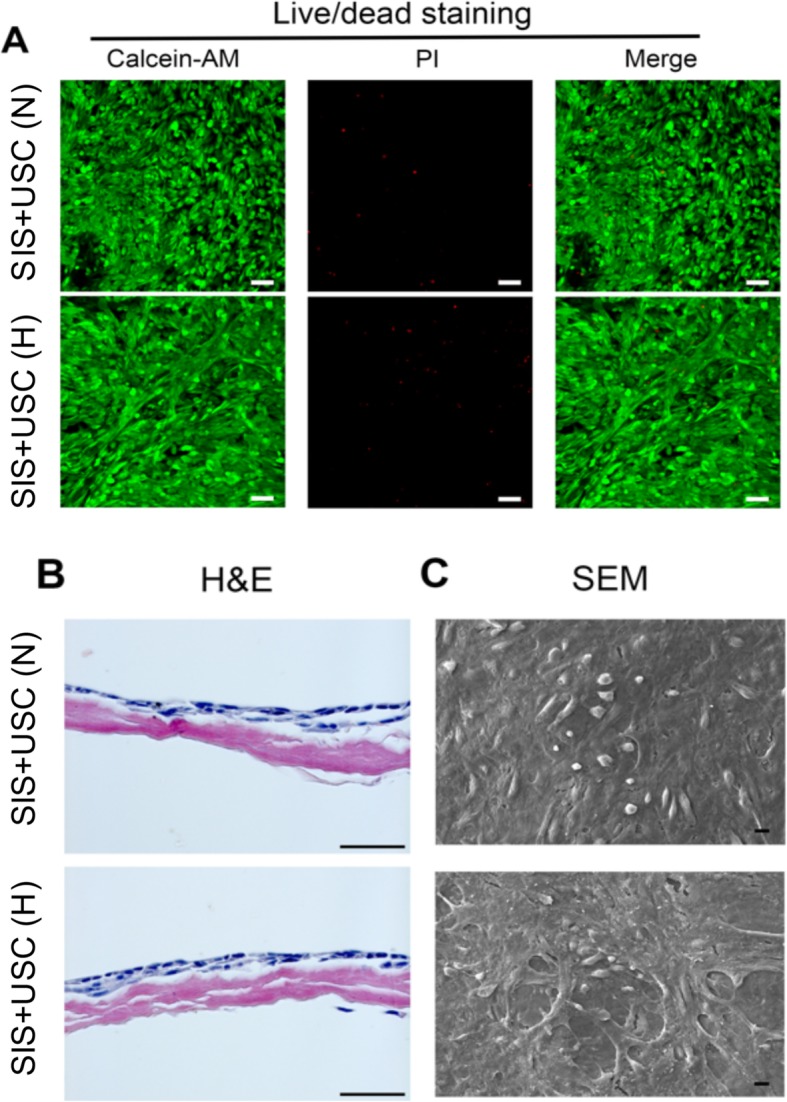
Viability and proliferation of USCs seeded on SIS membranes. **a** Live/dead staining of the SIS+USC composites cultured in normoxia (i.e., the SIS+USC (N) group) or preconditioned in hypoxia (i.e., the SIS+USC (H) group). Green fluorescence, live cells; red fluorescence, dead cells; scale bar, 50 μm. **b** H&E staining of the SIS+USC composites. Scale bar, 50 μm. **c** SEM observation of the SIS+USC composites. Scale bar, 10 μm

Twenty-four hours after cell seeding, a good cell viability was recorded in the SIS+USC (N) and SIS+USC (H) groups (Fig. 2a). USCs formed a multilayer cell sheet in both groups (Fig. 2b), and according to SEM observation (Fig. 2c), the cells spread well with a flattened morphology.

**Fig. 3:**
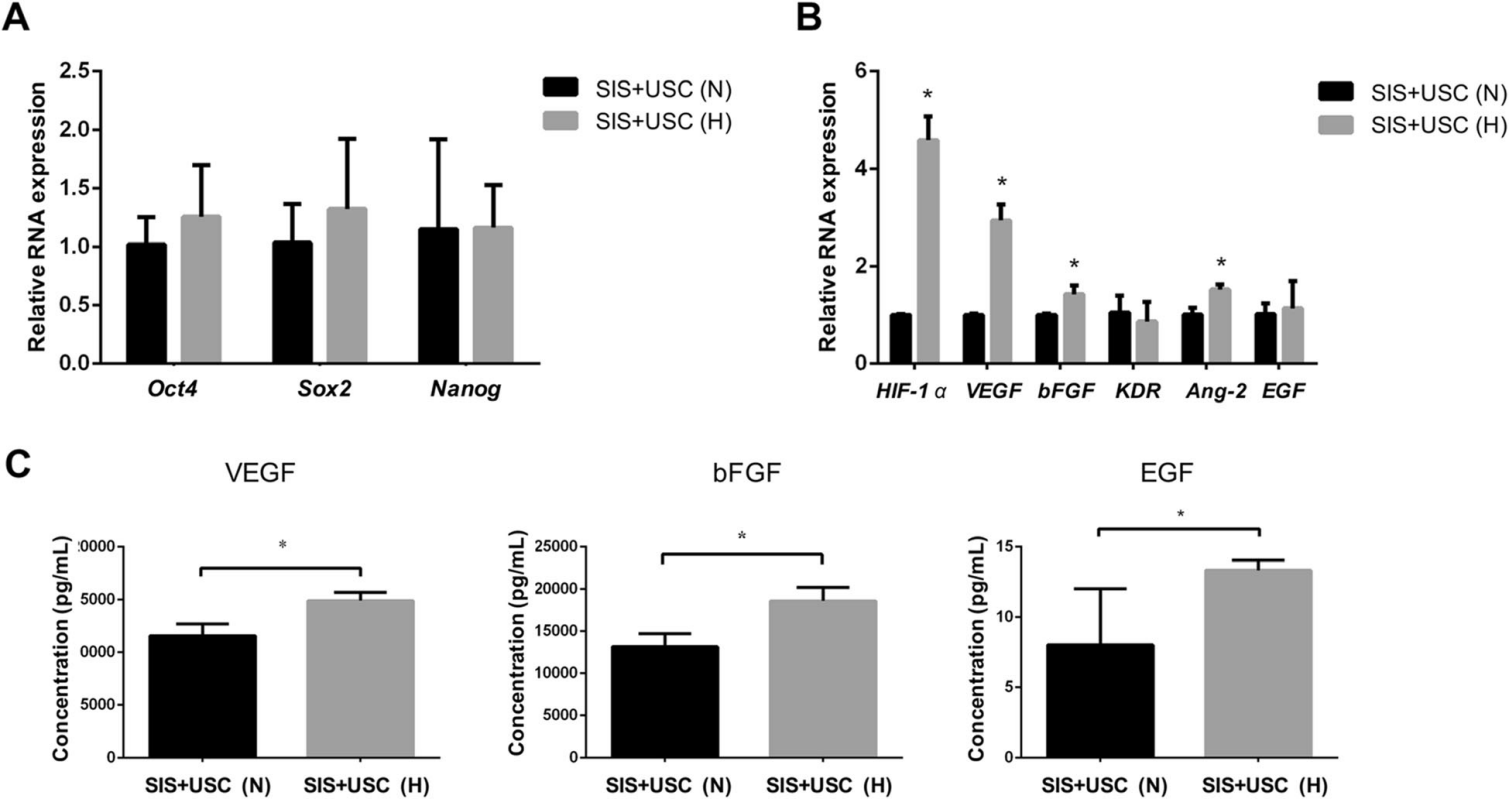
Gene expression and growth factor secretion of the SIS+USC composites. **a**, **b** Gene expression of the SIS+USC composites; *p < 0.05. **c** Growth factor secretion of the SIS+USC composites; *p < 0.05

To explore whether hypoxic preconditioning influenced the gene expression of USCs related to cell stemness, angiogenesis, and reepithelialization, RT-PCR was performed to assess the gene expression of *NANOG*, *SOX*-*2*, *Oct*-*4*, *HIF*-*1*, *VEGF*, *KDR*, *Ang*-*2*, *bFGF*, and *EGF* in the SIS+USC (N) and SIS+USC (H) groups. The results showed that hypoxic preconditioning did not change the expression of stemness factors, including *NANOG*, *SOX*-*2*, and *Oct*-*4* (Fig. 3a). However, the expression of genes related to angiogenesis (*VEGF* and *Ang*-*2*) and reepithelialization (*bFGF* and *EGF*) were obviously enhanced in the hypoxic preconditioning group (Fig. 3b). Additionally, the SIS+USC (H) group showed more secretion of growth factors (VEGF, EGF, and bFGF) than the SIS+USC (N) group (Fig. 3c).

### Hypoxia preconditioning enhances the wound healing potential of the SIS+USC composites

#### Gross observation

**Fig. 4:**
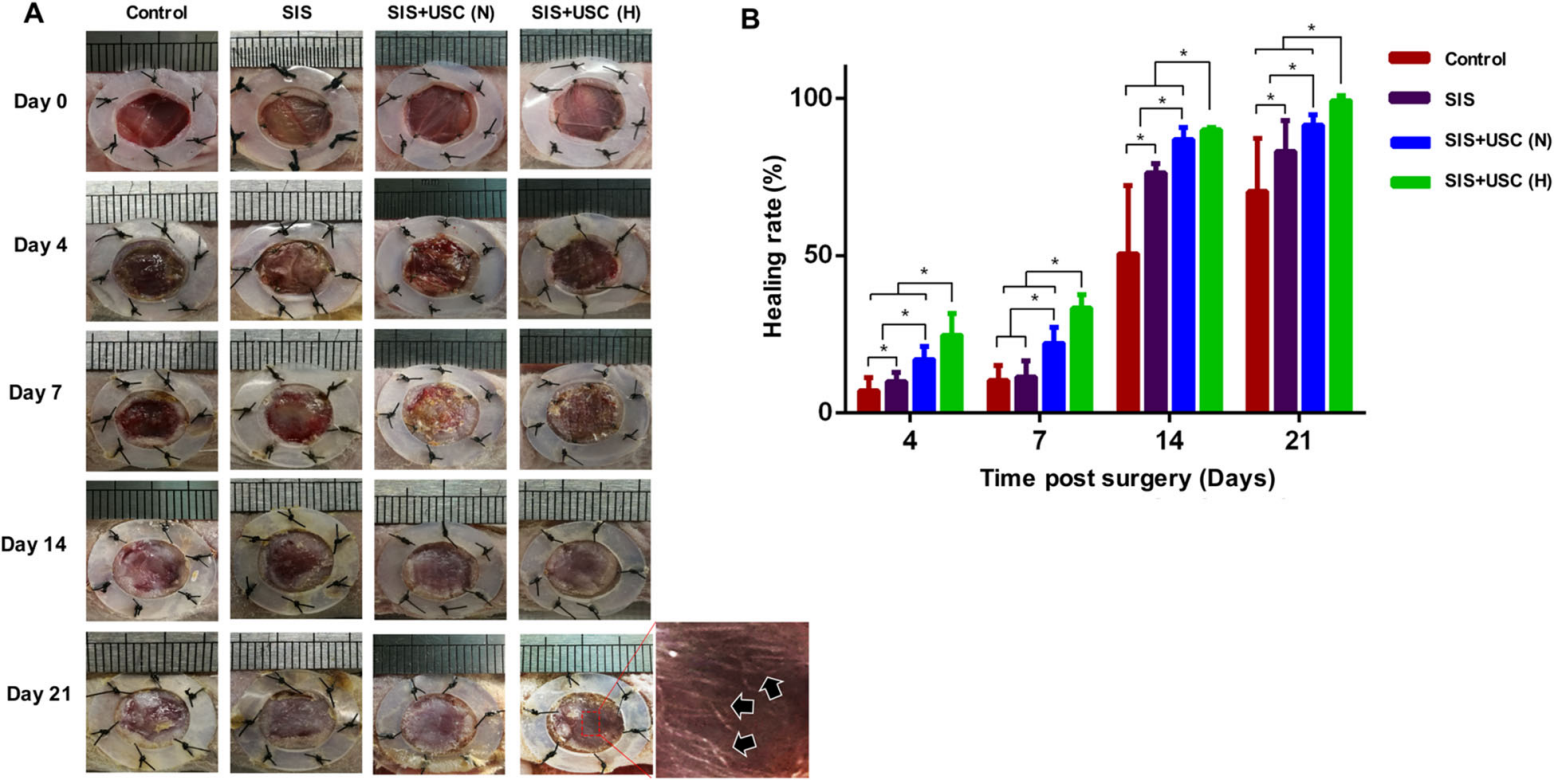
Macroscopic analysis of wound healing. **a** Gross view of the wounds; black arrows indicate regenerated hair in the wound area. **b** The healing rate of wounds in all groups; *p < 0.05

At predetermined time points, all wounds from each group were photographed to evaluate the wound healing rate. As shown in Fig. 4a, the wounds in the SIS+USC (H) group healed faster than that in other groups. Interestingly, in the SIS+USC (H) group, some new hair was observed at the center of the wound beds on day 21.

Comparing with the SIS group, a combination of SIS and USCs resulted in a higher healing rate and particularly, a superior healing rate was observed in the SIS+USC (H) group when compared with the SIS+USC (N) group (Fig. 4b).

#### Granulation tissue formation

**Fig. 5:**
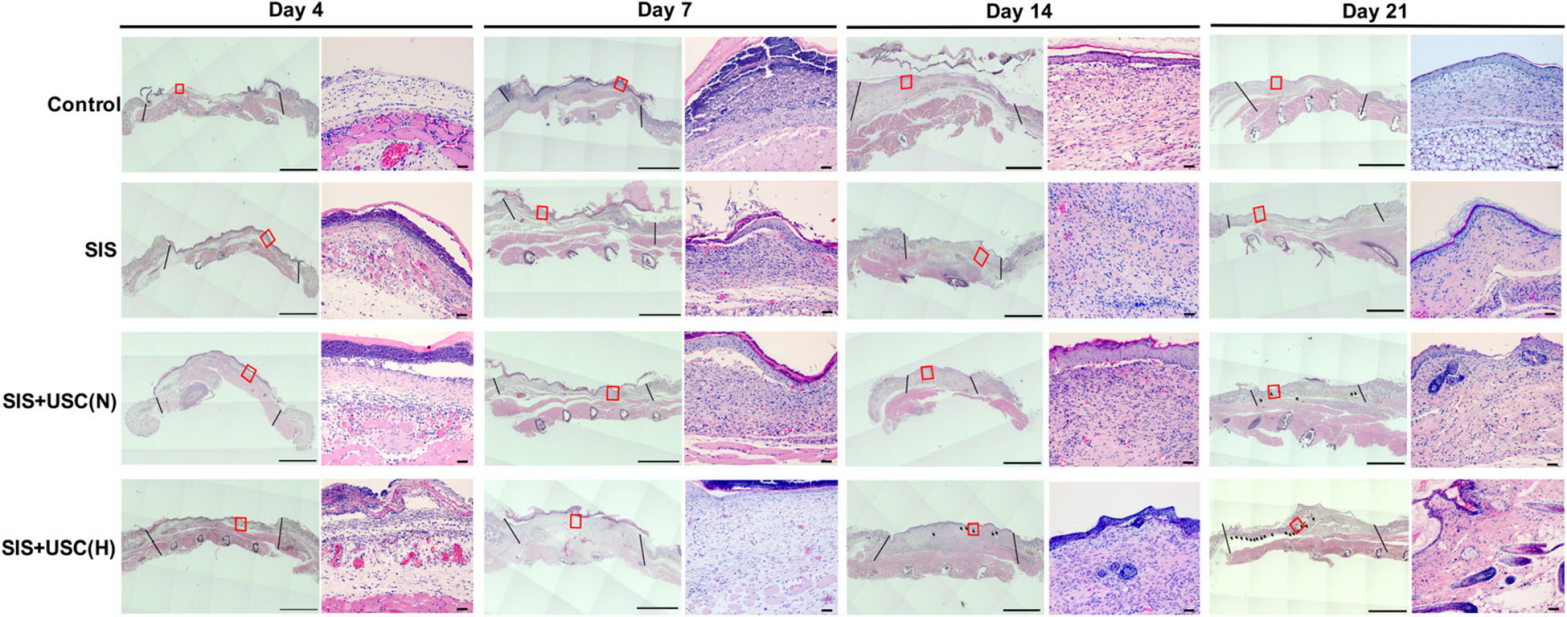
Representative images of H&E staining of the wounds. Black lines indicate the boundaries of wound healing areas. Black arrows indicate new skin appendages. Red rectangles represent the corresponding magnification images on the right column of images at each time point. Scale bar in the left column of images at each time point is 2000 μm. Scale bar in the right column of images at each time point is 50 μm

H&E staining was performed to observe granulation tissue formation in each group (Fig. 5). The H&E staining of normal nude mouse skin is shown in Additional file 2: Figure S1A.

On day 4 after implantation, continuous granulation tissue with plenty of inflammatory cells was noted in each group; interestingly, the control group did not form any clots, which was consistent with the results of gross observation (Fig. 4a).

On day 7, all groups formed a clot and the number of fibroblasts and deposition of extracellular matrix increased greatly. On day 14, some skin appendages were observed in the SIS+USC (H) group, while they were absent in other groups (Fig. 5).

On day 21, more skin appendages were observed in the SIS+USC (H) group and some new skin appendages were also noted in the SIS+USC (N) group. However, in the control group and the SIS group, no skin appendages were observed (Fig. 5).

#### Neovascularization

To determine whether hypoxic preconditioning enhanced the neovascularization of the SIS+USC composites in vivo, CD31 staining was conducted.

**Fig. 6:**
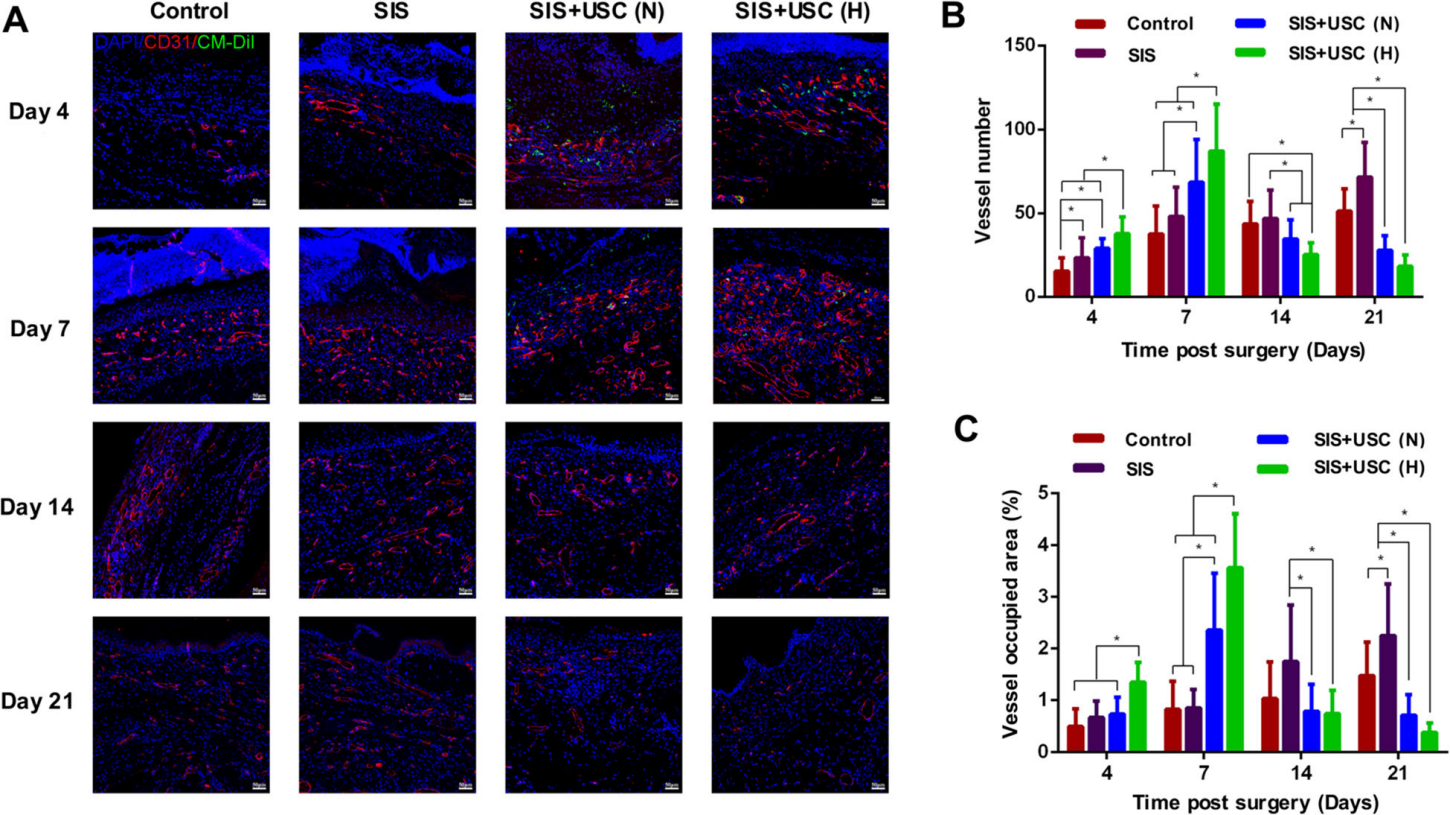
Neovascularization of the wounds. **a** Immunofluorescence staining. The blood vessels were stained with CD31 (red), the nucleus was stained with DAPI (blue), and the implanted USCs were stained with CM-Dil (green). Scale bar, 50 μm. **b** Quantitative analysis of the number of blood vessels; *p < 0.05. **c** Quantitative analysis of the rate of vessel occupied area; *p < 0.05

On days 4 and 7, new blood vessels in the SIS+USC (H) and SIS+USC (N) groups were more abundant and more mature than that of the control and SIS groups, featuring bigger lumen structures (Fig. 6a). On days 14 and 21, blood vessels in the SIS and control groups became more mature, as seen by more intact vessel walls and bigger lumens (Fig. 6a).

The detection of CM-Dil dye was performed to track the fate of labeled USCs in vivo. The results showed that USCs distributed in the wound bed on days 4 and 7 but only a few of them participated in vascular formation (Fig. 6a). However, from day 14 onwards, no labeled USCs were observed in these groups.

Quantitative analysis of the number of blood vessels and their occupied areas revealed that hypoxic preconditioning significantly enhanced the angiogenesis of the SIS+USC composites at an early stage of wound healing (Fig. 6b, c). In the SIS+USC (H) and SIS+USC (N) groups, the blood vessel numbers and vessel occupied areas achieved the highest levels on day 7 and then decreased gradually as the vessels matured. In contrast, in the control and SIS groups, neovascularization continued to increase on days 14 and 21 (Fig. 6b, c). On day 21, when comparing with other groups, the vascularization of the SIS+USC (H) group (vessel number 18.33 ± 6.76, vessel occupied area rate 0.38 ± 0.18%) was closer to that of normal nude mouse skin tissue (vessel number 17.32 ± 2.33, vessel occupied area rate 0.28 ± 0.10%, Additional file 2: Figure S1B–D).

#### Reepithelialization

Reepithelialization is essential to restore the elasticity and strength of epidermis in skin wounds [12]. In normal nude mouse skin, both of the epithelium and skin appendages are positive for CK14 staining (Additional file 2: Figure S1E) [32]; thus, this marker is used to evaluate the reepithelialization of wounds.

**Fig. 7:**
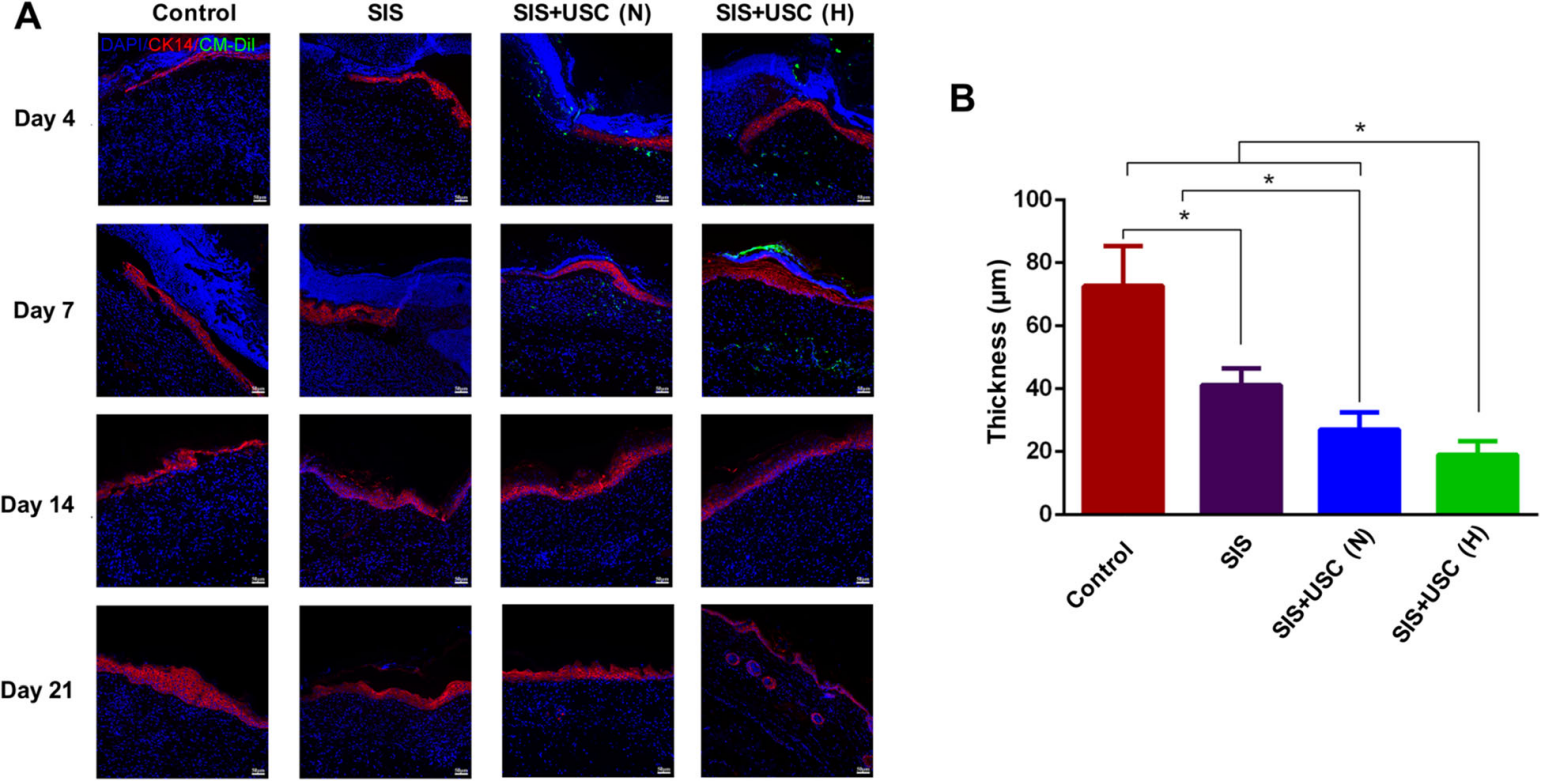
Reepithelialization of the wounds. **a** Immunofluorescence staining. The epithelia were stained with CK14 (red), the nucleus was stained with DAPI (blue), and USCs were stained with CM-Dil (green). Scale bar, 50 μm. **b** Quantitative analysis of the epithelia thickness on day 21 post-surgery; *p < 0.05

As shown in Fig. 7a, complete reepithelialization was achieved on day 14 in all groups. The SIS+USC (H) group showed better reepithelialization than other groups, as shown by more epithelial cell layers and better organization of the epidermis. On day 21, the epithelium thickness of the SIS+USC (H) group (19.0 ± 4.2 μm) was closest to normal nude mouse skin (19.6 ± 4.2 μm, Additional file 2: Figure S1F) (Fig. 7b). Interestingly, grafted USCs did not differentiate into epithelial cells, and they disappeared on days 14 and 21 (Fig. 7a).

#### Collagen deposition and remodeling

As a critical parameter of the quality of wound healing [33], the deposition of collagen fibers was determined by Sirius red staining. Due to the birefringence property under polarized light, type I collagen showed a red or yellow color, while type III collagen showed a green color [34]. In normal nude mouse skin tissue, mature collagen fibers formed a densely packed and basket weave-like network (Additional file 2: Figure S1G).

**Fig. 8:**
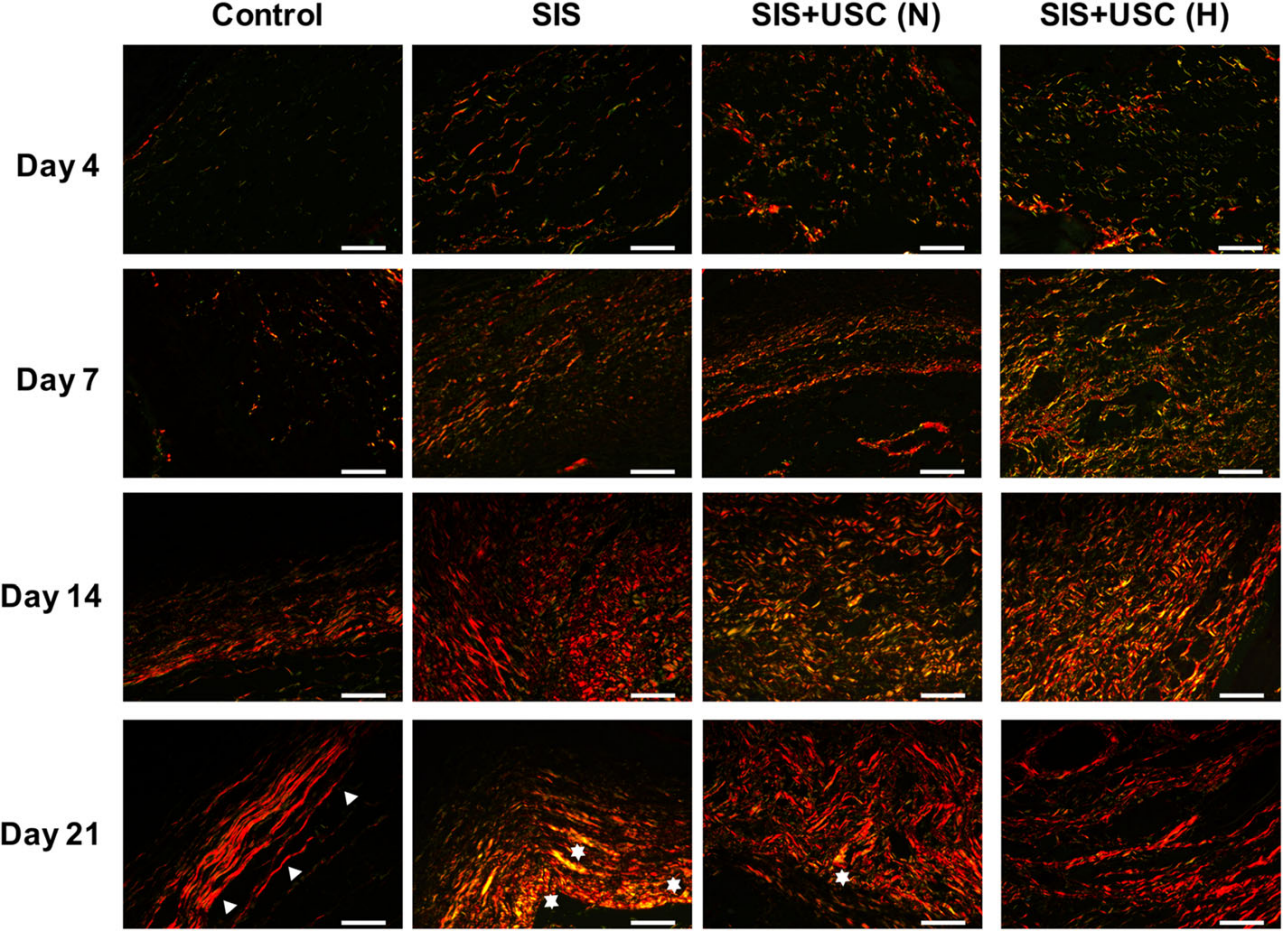
The structure of collagen fibers deposited in the wounds. White triangles represent flatter collagen fibers arranged in a parallel wavy pattern. White stars represent nodule collagen fibers. Scale bar, 50 μm

In this study, all groups deposited immature collagen fibers (fragmentary, thin, and loosely packed) on days 4 and 7; however, more collagen was noted in the SIS+USC (H) group than in other groups (Fig. 8). On day 14, a mixture of mature and immature collagen fibers was recorded in all groups, while on day 21, collagen fibers in the control group were arranged in a parallel wavy pattern, which was similar to a hypertrophic scar [35]. In the SIS and SIS+USC (N) groups, collagen fibers remodeled into a nodule-like structure that was markedly different from normal skin. In contrast, in the SIS+USC (H) group, mature collagen fibers with structures similar to normal skin were observed (Fig. 8).

## Discussion

Stem cell-based therapy holds great potential for treating difficult-to-heal skin wounds [36]. In this study, we created a tissue-engineered skin graft, termed the SIS+USC composite, by using a combination of a relatively new type of adult stem cell (i.e., USCs) and a widely used biomaterial for soft tissue repair (i.e., SIS membranes). The effect of hypoxic preconditioning on its wound healing potential was investigated in vitro and in vivo. According to our results, the SIS+USC composites showed superior wound healing outcomes when comparing with SIS alone. Notably, hypoxic preconditioning obviously enhanced the wound healing potential of the SIS+USC composite, as evidenced by the enhanced secretion of growth factors, accelerated neovascularization, facilitated reepithelialization, and promoted skin appendage regeneration in vivo.

Although stem cells have been considered beneficial for wound healing, their therapeutic effect is always impeded by poor cell survival at the wounds. It has been reported that 90% of grafted cells die on the first day after implantation, and less than 1% of transplanted cells remain detectable after a short period of time [37, 38]. In this study, the number of USCs at the wounds decreased dramatically after implantation, with grafted cells disappearing on day 14 and afterwards. Therefore, it is generally believed that the paracrine effect of cells, rather than their differentiation, is the main underlying repair mechanism for stem cell-based therapy [39, 40]. As such, several cell-free methods using the secretion of MSCs, such as their condition medium [41, 42] or exosomes [43, 44], have been introduced to treat skin wounds. Chen et al. demonstrated that exosomes derived from human USCs can effectively improve skin cell functions in vitro and obviously enhanced wound healing in diabetic mice [8].

Hypoxic preconditioning has been proposed to improve the therapeutic potential of stem cells, mainly by increasing their paracrine activities [45]. It has been reported that hypoxic conditioned medium from bone marrow- or amniotic fluid-derived MSCs accelerated skin wound healing in mice, primarily because of significant higher amounts of growth factors in the condition medium [41, 46]. Following hypoxia pretreatment, Tong et al. observed that bone marrow-derived MSCs seeded in a collagen-chitosan sponge scaffold upregulated their expression of pro-angiogenic factors; furthermore, hypoxia pretreatment of the skin substitute accelerated wound healing in diabetic rats [47]. Similarly, in this study, hypoxic preconditioning of the SIS+USC composite upregulated the expression of growth factors. More importantly, in vivo results revealed that the SIS+USC (H) group exhibited better neovascularization, reepithelialization, collagen deposition, and skin appendage regeneration than other groups.

The formation of new blood vessels is crucial to wound healing, because it is responsible for nutrient diffusion and the removal of metabolic waste [48–50]. MSCs have been widely regarded as a promising tool to enhance wound angiogenesis [51]. As a relatively new type of adult stem cells, USCs share a great deal of similarity with MSCs, thereby attracting considerable interest in the potential application for therapeutic angiogenesis. Previous studies have shown that USCs can secrete pro-angiogenetic factors [52–54], stimulate the proliferation of endothelial cells [52, 55], and differentiate into functional endothelial cells in vitro and in vivo [56].

When comparing with the untreated wounds, the injection of USCs suspension greatly improved skin wound healing by promoting angiogenesis [55, 57]. Interestingly, a combination of USCs with scaffolds, such as polycaprolactone/gelatin (PCL/GT) nanofibrous membranes or bacterial cellulose membranes, has showed better wound healing than that of the cell only or the scaffold only groups [9, 57]. In this study, we observed that USCs seeded on SIS membranes secreted a large amount of angiogenic factors, of which the expression was further stimulated by hypoxic preconditioning. Therefore, the SIS+USC (H) group showed the best vascularization at the early stage of wound healing, and this group resulted in a more rapid wound closure than other groups.

Upon skin injury, keratinocytes at the wound edge will receive the damage signals and reepithelialize the wounds [58, 59]. As a common response to dermal wounds, the thickness of epidermis increases at the early phase of wound healing, which is characterized by overdevelopment of epithelial cell layers [60]. The ability of USCs to improve wound reepithelialization has been observed in animal studies [8, 9, 55, 57]. For instance, Fu et al. reported that transplantation of USCs loaded PCL/GT membranes showed enhanced wound reepithelialization as compared with the PCL/GT membrane group [9]. Likewise, Cao et al. demonstrated that treatment with a combination of USCs and surface-structured bacterial cellulose membranes resulted in thicker and better arranged neonatal epithelium than the membrane-only group [57]. In this study, we found that hypoxic preconditioning of the SIS+USC composites enhanced the secretion of growth factors favoring wound reepithelialization in vitro. Notably, at the early stage of wound healing, the SIS+USC (H) group showed more epithelial cell layers than other groups. This might also be due to better angiogenesis in the SIS+USC (H) group, because sufficient nutrients are necessary for the regeneration of a new and healthy epidermis.

The dermis of rodent skin is mainly composed of types I and III collagen and elastic fibers [61]. It is well known that collagen fibers of normal skin are different from that of scar tissue. According to our results, except for the SIS+USC (H) group, none of the repair tissues showed collagen deposition similar to normal skin. Instead, they demonstrated collagen fibers similar to hypertrophic scar tissue [35, 62–64]. These results suggest that hypoxic preconditioning of the SIS+USC composite improved the quality of collagen deposition at the wounds.

Furthermore, skin appendages, such as hair follicles, sweat glands, and sebaceous glands, are critical for the function of normal skin. In this study, new skin appendages were first observed in the SIS+USC (H) group on day 14. Afterwards, they were also observed in the SIS+USC (N) group on day 21. These results may be attributed to the improved neovascularization at the early stage of wound healing [65]. Interestingly, USCs did not differentiate into skin appendages, indicating the crucial role of endogenous cells in the regeneration of skin appendages.

## Conclusions

This study demonstrates that hypoxic preconditioning of the SIS+USC composite enhances its wound healing potential. The secretion of growth factors related to angiogenesis and reepithelialization was greatly increased after hypoxic preconditioning. Compared with the SIS+USC (N) group, the SIS+USC (H) group showed better neovascularization at the early stage of wound healing, more abundant and earlier regeneration of skin appendages, faster wound closure, and better deposition of collagen fibers. Taken together, the SIS+USC composite holds great potential for skin wound healing, and importantly, hypoxic preconditioning provides a simple and effective method to improve its wound healing potential.

## Supplementary information

**Additional file 1: Table S1.** Primers for RT-PCR.

**Additional file 2: Figure S1.** Histology of normal nude mouse skin. (A) H&E staining. (B) CD31 immunofluorescence staining. (C) The occupied area rate of vessels in normal nude mouse skin was 0.28 ± 0.10%. (D) The number of blood vessels per field in normal nude mouse skin was 17.32 ± 2.33. (E) CK14 immunofluorescence staining. (F) The thickness of epithelia in normal nude mouse skin was 19.6 ± 4.2 μm. (G) Sirius red staining.

## Data Availability

All data generated or analyzed during this study are included in this published article and its supplementary information files.

## Abbreviations

bFGF: Basic fibroblast growth factor
EGF: Epidermal growth factor
ELISA: Enzyme-linked immunosorbent assay
H&E: Hematoxylin and eosin
MSCs: Mesenchymal stem cells
PBS: Phosphate-buffered saline
PFA: Paraformaldehyde
SIS: Small intestine submucosa
RT-PCR: Real-time polymerase chain reaction
SEM: Scanning electron microscope
USCs: Urine-derived stem cells
VEGF: Vascular endothelial growth factor
