# Supplementary material for: Hypoxic preconditioning of human urine-derived stem cell-laden small intestinal submucosa enhances wound healing potential
Source: Stem Cell Res Ther. 2020 Apr 6;11:150. doi: 10.1186/s13287-020-01662-2 (PMC7137341; doi:10.1186/s13287-020-01662-2)
Supplement: Supplementary file 1 — Additional file 1: Table S1. Primers for RT-PCR. [file 13287_2020_1662_MOESM1_ESM.docx]

**Table S1 Primers for RT-PCR**

| Genes | Forward primer (5’-3’) | Reverse primer (5’-3’) |
| --- | --- | --- |
| *GAPDH* | GGAGCGAGATCCCTCCAAAAT | GGCTGTTGTCATACTTCTCATGG |
| *Oct-4* | CTTGAATCCCGAATGGAAAGGG | CCTTCCCAAATAGAACCCCCA |
| *NANOG* | CCCCAGCCTTTACTCTTCCTA | CCAGGTTGAATTGTTCCAGGTC |
| *SOX-2* | TACAGCATGTCCTACTCGCAG | GAGGAAGAGGTAACCACAGGG |
| *HIF-1**α* | ATCCATGTGACCATGAGGAAATG | TCGGCTAGTTAGGGTACACTTC |
| *VEGF* | AGGGCAGAATCATCACGAAGT | AGGGTCTCGATTGGATGGCA |
| *KDR* | GGCCCAATAATCAGAGTGGCA | CCAGTGTCATTTCCGATCACTTT |
| *Ang-2* | AACTTTCGGAAGAGCATGGAC | CGAGTCATCGTATTCGAGCGG |
| *bFGF* | AGAAGAGCGACCCTCACATCA | CGGTTAGCACACACTCCTTTG |
| *EGF* | TGTCCACGCAATGTGTCTGAA | CATTATCGGGTGAGGAACAACC |

bFGF: basic fibroblast growth factor; EGF: epidermal growth factor; VEGF: vascular endothelial growth factor.
