# Supplementary material for: Hypoxic preconditioning of human urine-derived stem cell-laden small intestinal submucosa enhances wound healing potential
Source: Stem Cell Res Ther. 2020 Apr 6;11:150. doi: 10.1186/s13287-020-01662-2 (PMC7137341; doi:10.1186/s13287-020-01662-2)
Supplement: Supplementary file 2 — Additional file 2: Figure S1. Histology of normal nude mouse skin. (A) H&E staining. (B) CD31 immunofluorescence staining. (C) The occupied area rate of vessels in normal nude mouse skin was 0.28 ± 0.10%. (D) The number of blood vessels per field in normal nude mouse skin was 17.32 ± 2.33. (E) CK14 immunofluorescence staining. (F) The thickness of epithelia in normal nude mouse skin was 19.6 ± 4.2 μm. (G) Sirius red staining. [file 13287_2020_1662_MOESM2_ESM.docx]

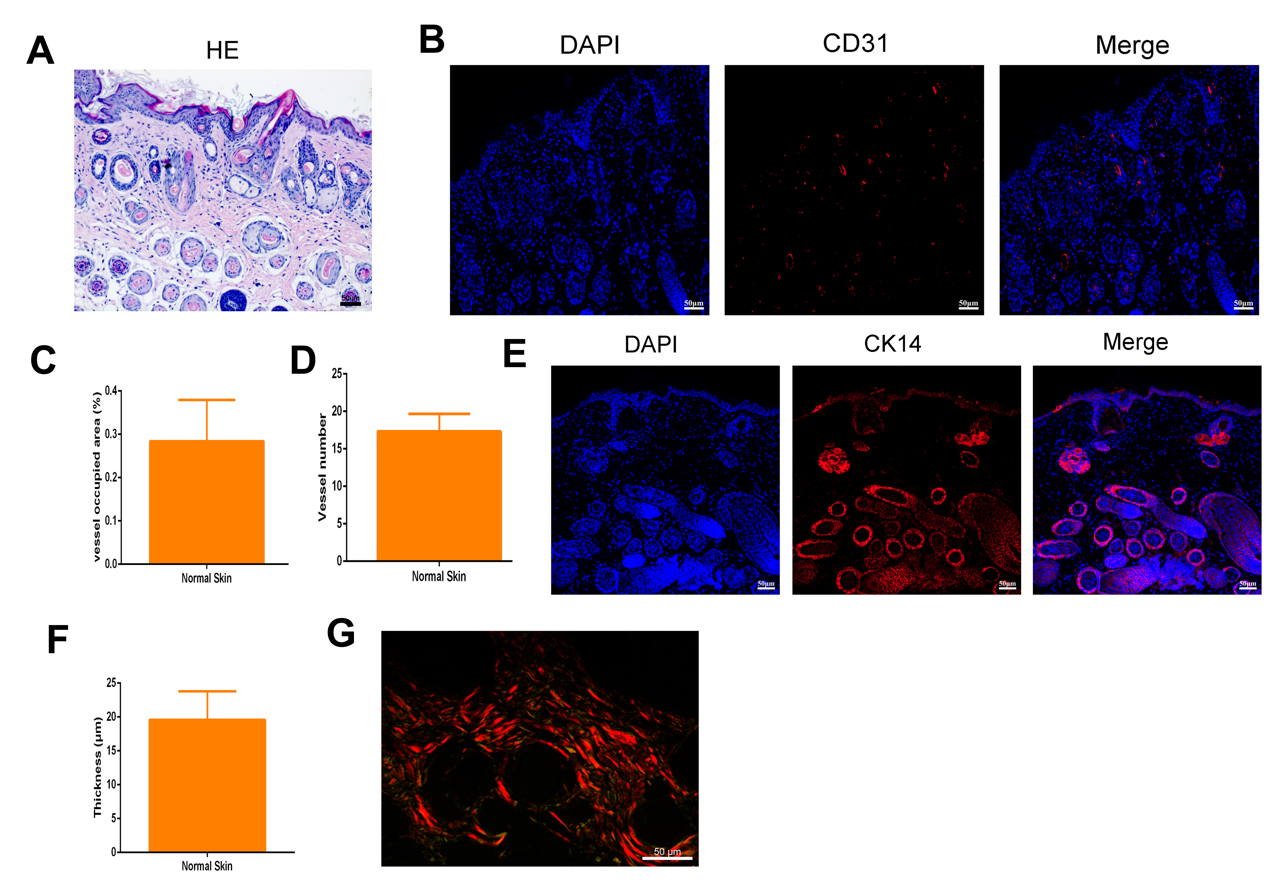


**Figure S1. Histology of normal nude mouse skin.** (A) H&E staining. (B) CD31 immunofluorescence staining. (C) The occupied area rate of vessels in normal nude mouse skin was 0.28 ± 0.10%. (D) The number of blood vessels per field in normal nude mouse skin was 17.32 ± 2.33. (E) CK14 immunofluorescence staining. (F) The thickness of epithelia in normal nude mouse skin was 19.6 ± 4.2 μm. (G) Sirius red staining.
